# Results of a diagnostic imaging audit in a randomised clinical trial in rectal cancer highlight the importance of careful planning and quality control

**DOI:** 10.1186/s13244-023-01552-0

**Published:** 2023-11-24

**Authors:** Ilaria Prata, Martina Eriksson, Jasenko Krdzalic, Elma Meershoek-Klein Kranenbarg, Annet G. H. Roodvoets, Regina Beets-Tan, Cornelis J. H. van de Velde, Boudewijn van Etten, Geke A. P. Hospers, Bengt Glimelius, Per J. Nilsson, Corrie A. M. Marijnen, Koen C. M. J. Peeters, Lennart K. Blomqvist

**Affiliations:** 1 Department of Surgery, Netherlands Cancer Institute, Amsterdam, the Netherlands; 2 Department of Surgery, Leiden University Medical Center, Leiden, the Netherlands; 3 GROW School for Oncology and Reproduction, Maastricht University, Maastricht, the Netherlands; 4 Department of Radiology, Capio S:T Göran Hospital, Stockholm, Sweden; 5 Department of Radiology, Zuyderland Medical Center, Geleen, the Netherlands; 6 Department of Radiology, Netherlands Cancer Institute, Amsterdam, the Netherlands; 7 Department of Surgery, University Medical Center Groningen, Groningen, the Netherlands; 8 Department of Medical Oncology, University Medical Center Groningen, Groningen, the Netherlands; 9 Department of Immunology, Genetics and Pathology, Uppsala University, Uppsala, Sweden; 10 Department of Pelvic Cancer, Karolinska University Hospital, Stockholm, Sweden; 11 Department of Radiation Oncology, Netherlands Cancer Institute, Amsterdam, the Netherlands; 12 Department of Radiation Physics/Nuclear Medicine, Karolinska University Hospital, Stockholm, Sweden

**Keywords:** Rectal cancer, Magnetic resonance imaging, Image acquisition protocol, Audit

## Abstract

**Background:**

Magnetic resonance (MR) imaging is the modality used for baseline assessment of locally advanced rectal cancer (LARC) and restaging after neoadjuvant treatment. The overall audited quality of MR imaging in large multicentre trials on rectal cancer is so far not routinely reported.

**Materials and methods:**

We collected MR images obtained within the Rectal Cancer And Pre-operative Induction Therapy Followed by Dedicated Operation (RAPIDO) trial and performed an audit of the technical features of image acquisition. The required MR sequences and slice thickness stated in the RAPIDO protocol were used as a reference.

**Results:**

Out of 920 participants of the RAPIDO study, MR investigations of 668 and 623 patients in the baseline and restaging setting, respectively, were collected. Of these, 304/668 (45.5%) and 328/623 (52.6%) MR images, respectively, fulfilled the technical quality criteria. The main reason for non-compliance was exceeding slice thickness 238/668, 35.6% in the baseline setting and 162/623, 26.0% in the restaging setting. In 166/668, 24.9% and 168/623, 27.0% MR images in the baseline and restaging setting, respectively, one or more of the required pulse sequences were missing.

**Conclusion:**

Altogether, 49.0% of the MR images obtained within the RAPIDO trial fulfilled the image acquisition criteria required in the study protocol. High-quality MR imaging should be expected for the appropriate initial treatment and response evaluation of patients with LARC, and efforts should be made to maximise the quality of imaging in clinical trials and in clinical practice.

**Critical relevance statement:**

This audit highlights the importance of adherence to MR image acquisition criteria for rectal cancer, both in multicentre trials and in daily clinical practice. High-resolution images allow correct staging, treatment stratification and evaluation of response to neoadjuvant treatment.

**Key points:**

- Complying to MR acquisition guidelines in multicentre trials is challenging.

- Neglection on MR acquisition criteria leads to poor staging and treatment.

- MR acquisition guidelines should be followed in trials and clinical practice.

- Researchers should consider mandatory audits prior to study initiation.

**Graphical Abstract:**

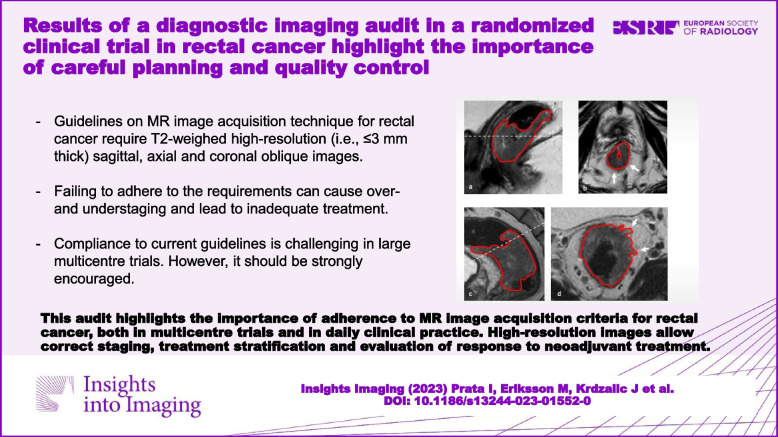

**Supplementary Information:**

The online version contains supplementary material available at 10.1186/s13244-023-01552-0.

## Introduction

Colorectal cancer is the third most common cancer in the world, accounting for 10% of all cancers [1]. The proportion of rectal cancer varies depending on the classification used and usually accounts for one third of all colorectal cancers [1, 2]. Accurate staging of rectal cancer is important because treatment and prognosis depend largely on radiological classification. Historically, staging of rectal cancer was done using only digital examination and rectoscopy. Currently, magnetic resonance imaging (MRI) is the technique of choice for local staging of rectal cancer, both at baseline and as reassessment after neoadjuvant treatment for locally advanced rectal cancer [3, 4]. Accurate (re)staging is of utmost importance for assigning patients to the most appropriate treatment. Additionally, MRIs performed after neoadjuvant treatment contribute to the referral of patients for non-operative management [5].

Although MRI is considered the optimal local staging technique for rectal cancer, there are still challenges. The image quality and evaluation are of paramount importance since consistent high quality is required to make a correct analysis of the tumour spread [6, 7]. Quality has an impact both clinically for each patient but also in the setting of a clinical trial to ensure that patients are correctly stratified to treatment according to stipulated inclusion criteria [6, 7]. Therefore, correct, standardised MRI protocols should be used and followed [8].

The Rectal Cancer And Pre-operative Induction Therapy Followed by Dedicated Operation (RAPIDO) trial is an international randomised controlled phase 3 trial [9]. In the scope of the study, pelvic MRI was performed at initial staging and after neoadjuvant treatment. Additionally, a pelvic MRI was recommended during neo-adjuvant treatment. In the study protocol, there were clear quality requirements referring to the MRI acquisition protocol. This retrospective study aimed to evaluate whether MRIs performed during the RAPIDO trial fulfilled the quality requirements regarding image acquisition stated in the study protocol. Moreover, a comparison with the quality criteria for MRI in other randomised controlled trials (RCT) for locally advanced rectal cancer was performed.

## Materials and methods

In the RAPIDO trial, patients with primary locally advanced rectal cancer defined by high-risk features on MRI evaluation were randomised between two different neoadjuvant treatment regimens followed by surgery according to the principles of total mesorectal excision (TME). Participants allocated to the experimental group received short-course radiotherapy followed by fluorouracil- and oxaliplatin-based chemotherapy. Patients allocated to the standard of care group received long course chemo-radiotherapy with concomitant capecitabine. Inclusion criteria, neoadjuvant treatment schedules and endpoints have been reported previously [9].

### MRI protocol requirements

Each included patient underwent one baseline pelvic MRI examination within 5 weeks before randomisation and a restaging pelvic MRI examination after neoadjuvant therapy. The following minimal requirements for MRI acquisition protocols applied to both the baseline and the reassessment investigations: a field strength of 1.5 T or 3 T and phased-array receiver coils for pelvic/body imaging, T2-weighted high-resolution sequences in three different planes (sagittal, axial and coronal oblique planes) with the axial sequence perpendicular to the tumour axis, with maximum 3 mm section thickness for all sequences (see Additional file 1: Supplementary Materials). In case of low tumours, additional oblique sequences both parallel and perpendicular to the anal canal were recommended. Additional sequences, such as T1-weighted and diffusion-weighted imaging (DWI) at restaging were highly recommended but not part of the obligatory pulse sequences [10]. These requirements comply with the most recent international consensus guidelines [4, 11, 12].

### Quality control

The intention to collect and centrally review the radiology data with the purpose of quality control was stated in the original protocol of the RAPIDO trial [10], accepted by all participants at the time of first patient inclusion. This audit has been performed retrospectively after the conclusion of the main trial. All patients whose MR images were evaluated in the context of this audit had been included in the study and treated according to the study protocol.

**Fig. 1:**
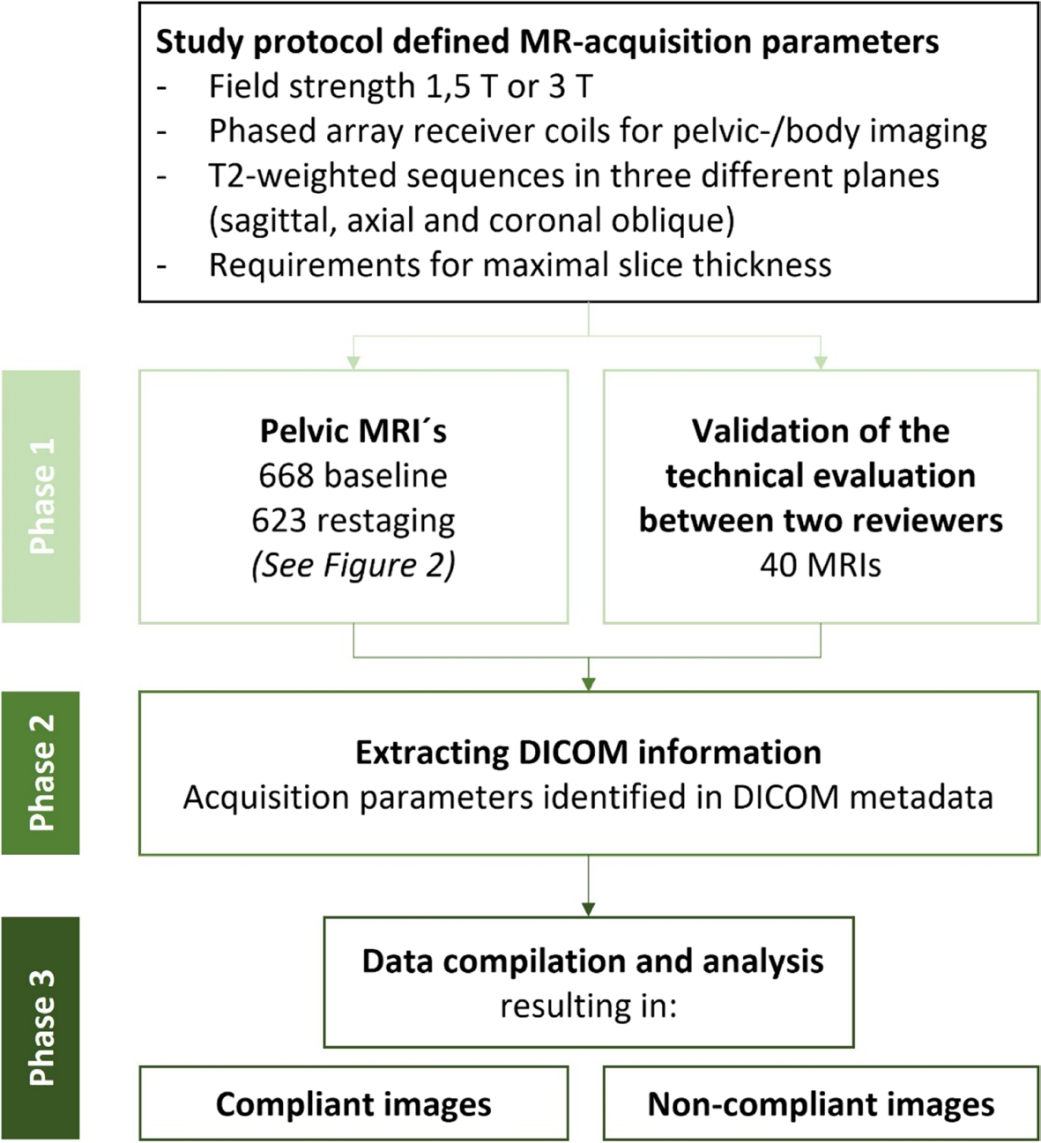
Process of image collection and review. DICOM = Digital Imaging and Communications in Medicine; MR(I) = Magnetic Resonance (Imaging)

Out of the 920 participants to the RAPIDO study, it was possible to retrieve MR images of the majority of Dutch and Swedish patients (*n* = 361 and 332 patients, respectively) and from all patients from Slovenia (*n* = 36) for the current study. Data were analysed both in the complete selection of patients and in national subgroups. All collected baseline and restaging MRI examinations were assessed for the technical quality criteria by two reviewers (M.E., I.P.). In particular, the presence of all required sequences and slice thickness were assessed for each investigation. To define a common method for evaluation, an initial sample of 40 investigations were assessed by both reviewers. The work was supervised by a radiologist with more than 30 years of experience in reviewing pelvic MRI (L.B.). For oblique high-resolution T2-weighted sequences perpendicular to the tumour, a slice thickness of up to 3.3 mm was regarded as acceptable. Moreover, a slice thickness of up to 4 mm on sagittal sequences was accepted, provided that all other required sequences were not thicker than 3 mm. The process of image revision is presented in Fig. 1.

### Review of similar articles

Recently published RCTs and European radiology guidelines were reviewed as a comparison with this quality audit performed on RAPIDO MRI examinations. A PubMed search using the search terms “magnetic resonance imaging” and “rectal cancer” combined with the limitations of Randomized Controlled Trials regarding human subjects and English language was performed and yielded 54 articles (January 2023). In addition, the most recent European Society of Gastrointestinal and Abdominal Radiology (ESGAR) guidelines were consulted as qualitative reference [4].

## Results

**Fig. 2:**
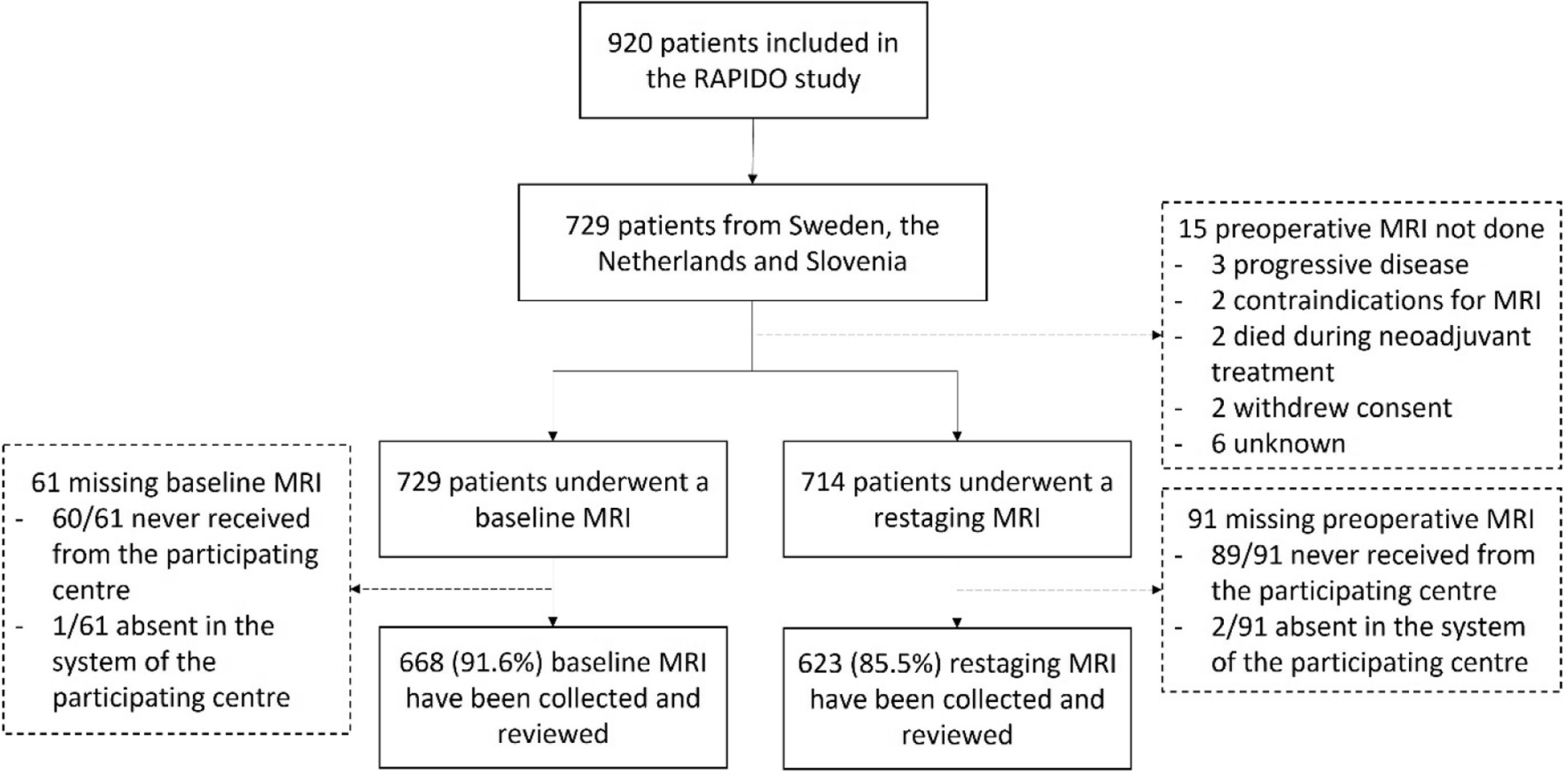
CONSORT diagram of the population considered for this audit. MRI = Magnetic Resonance Imaging; RAPIDO = Rectal Cancer And Pre-operative Induction Therapy Followed by Dedicated Operation

The MRI investigations of a total of 729/920 (79.2%) patients included in the RAPIDO trial were considered for this study. The MRI examinations of 668/729 (91.6%) and 623/729 (85.5%) patients were available for review in the baseline and restaging setting, respectively (Fig. 2). Most unavailable scans were not retrievable from the participating centres or absent in the systems where patients had been treated. Some patients were unavailable for radiologic assessment (died during neoadjuvant treatment (*n* = 2), clinical progression of disease before the time of reassessment (*n* = 3), contraindication to MRI (*n* = 2), withdrew consent to the study (*n* = 2) and unknown reason (*n* = 6)).

### Compliance to the protocol

In the baseline setting, 304/668 (45.5%) MRI examinations fulfilled the acquisition criteria stipulated in the protocol. The reasons for non-compliance to the protocol in the remaining 364 examinations were exceeding slice thickness of one or more sequences (90/668, 13.5% and 147/668, 22.0%, respectively) or absence of one or more of the required sequences (69/668, 10.3% and 97/668, 15.5%, respectively). In 40/668 (6.0%), both reasons occurred simultaneously.

In the restaging setting, 328/623 (52.6%) MRI examinations complied to the protocol. Of the 295/623 scans that did not fulfil the protocol, 162 exceeded slice thickness (75/623, 12.0% and 85/623, 13.7% for one or more sequences, respectively), and in 27.0% of cases, one or more of the required sequences were missing (88/623, 14.1% and 80/623, 12.8%, for a single and multiple sequence[s], respectively). In 5.6%, both reasons occurred simultaneously (35/623) as shown in Table 1.

**Table 1:** Number of MRI investigations fulfilling the pre-defined protocol criteria and the reasons for non- compliance in both the baseline and the preoperative settings. Numbers and percentages refer to the whole study group and to the country of each patient group

		Baseline *N*—%								Restaging *N*—%							
		Total *N* = 668		Sweden *N* = 302		Netherlands *N* = 331		Slovenia *N* = 35		Total *N* = 623		Sweden *N* = 281		Netherlands *N* = 306		Slovenia *N* = 36	
Fulfilling protocol criteria		304	45.5%	164	54.3%	116	35.0%	24	68.6%	328	52.6%	165	58.7%	127	41.5%	36	100%
*Fulfilling original protocol*		*232*	*34.7%*	*119*	*39.4%*	*91*	*27.4%*	*22*	*62.9%*	*272*	*43.7%*	*143*	*50.9%*	*93*	*30.4%*	*36*	*100%*
*Fulfilling adapted protocol*^a^		*72*	*10.8%*	*45*	*14.9%*	*25*	*7.6%*	*2*	*5.7%*	*56*	*9.0%*	*22*	*7.8%*	*34*	*11.1%*	*0*	*0%*
Not fulfilling protocol criteria		364	54.5%	138	45.7%	215	65%	11	31.4%	295	47.4%	116	41.3%	179	58.5%	0	0%
**Criteria fulfilment**																	
Slice thickness	Fulfilling^b^	430	64.4%	223	73.8%	180	54.4%	27	77.1%	461	74.0%	226	80.4%	199	65.0%	36	100%
	Not fulfilling	238	35.6%	78	26.2%	151	45.6%	8	22.9%	162	26.0%	55	19.6%	107	35.0%	0	0%
	*One sequence*	*90*	*13.5%*	*51*	*16.9%*	*34*	*10.3%*	*5*	*14.3%*	*75*	*12.0%*	*39*	*13.9%*	*36*	*11.8%*	*NA*	
	≥ *Two sequences*	*147*	*22.0%*	*28*	*9.3%*	*116*	*35.0%*	*3*	*8.6%*	*85*	*13.7%*	*16*	*5.7%*	*69*	*22.5%*	*NA*	
	*No sequences available*^c^	*1*	*0.1%*	*0*	*0.0%*	*1*	*0.3%*	*0*	*0.0%*	*2*	*0.3%*	*0*	*0.0%*	*2*	*0.7%*	*NA*	
Missing sequences	Fulfilling	502	75.1%	227	75.2%	243	73.4%	32	91.7%	455	73.0%	206	73.3%	213	69.6%	36	100%
	Not fulfilling	166	24.9%	75	24.8%	88	26.6%	3	8.6%	168	27.0%	75	26.7%	93	30.4%	0	0%
	*One sequence*	*69*	*10.3%*	*27*	*8.9%*	*39*	*11.8%*	*3*	*8.6%*	*88*	*14.1%*	*30*	*10.7%*	*58*	*19.0%*	*NA*	
	≥ *Two sequences*	*97*	*14.5%*	*48*	*15.9%*	*49*	*14.8%*	*0*	*0%*	*80*	*12.8%*	*45*	*16.0%*	*35*	*14.5%*	*NA*	

^a^All sequences up to 3.3 mm thickness and sagittal sequences only up to 4 mm

^b^Includes cases with sagittal sequences only up to 4 mm

^c^No sagittal, axial and coronal oblique sequences available

**Fig. 3:**
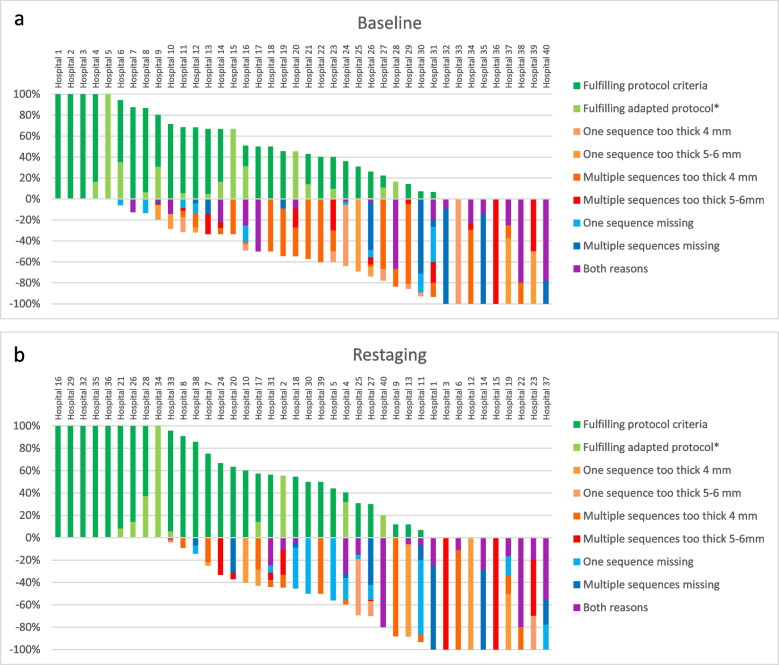
**a**, **b** Details of reasons for non-fulfilment per participating institution considered in this audit (**a**) in the baseline and (**b**) restaging setting. Reasons for non-fulfilment recur within most centres. Symbol “*” indicates the following: all sequences up to 3.3 mm thickness and sagittal sequences only up to 4 mm

For both the baseline and the restaging setting, the details of slice thickness protocol deviations are presented in Table 2. The number of MRIs with a 4-mm sagittal sequences and 3-mm axial and oblique sequences that were considered compliant for this analysis were 90/668 (13.5%) in the baseline setting and 55/623 (8.8%) in the restaging setting. Reasons for not fulfilment per participating centre are plotted in Fig. 3a and b for the baseline and the restaging settings, respectively. The figure shows groups of centres with a similar level of (non-) fulfilment of protocol criteria. Reasons for not complying to the protocol often recur within each centre.

**Table 2:** Details of slice thickness deviations

	Baseline *N*—%						Restaging *N*—%					
	Sagittal *N* = 666		Axial oblique *N* = 559		Coronal oblique *N* = 514		Sagittal *N* = 621		Axial oblique *N* = 529		Coronal oblique *N* = 472	
3–4 mm	220	33.0%	122	21.8%	117	22.8%	143	23.1%	88	16.6%	67	14.2%
5–6 mm	75	11.3%	22	3.9%	13	2.5%	56	9.1%	6	1.1%	4	0.8%

### Review of similar articles

Of the 54 articles identified during the literature search, 20 studies referred to patients with locally advanced rectal cancer and reported the results of a randomised clinical trials, including the RAPIDO trial. Together with the ESGAR guidelines, these studies and the corresponding MRI quality requirements as described in the main manuscript or in the Supplementary material, when published, are presented in Table 3. Among the selected RCTs, eleven (55%) explicitly referred to specific MRI quality requirements. Most commonly, the field strength of the machines used to perform the MRI investigations, the employment of DWI and the exact description of the required sequences were specified as requirements. Slice thickness ≤ 3 mm is mentioned in 4/20 studies (20.0%).

**Table 3:** English written randomised controlled trials regarding locally advanced rectal cancer and considering MRI as a (re)staging technique

	Study	Field strength	Phased-array receiver coils	T2W general	T2 sagittal	T2 axial	T2 perpendicular to long axis	T2 coronal	T2 parallel to long axis	DWI	Other description
	ESGAR guidelines 2018 [4]	1.5/3.0 T	Yes	Yes	Yes ≤ 3 mm		Yes ≤ 3 mm		Yes ≤ 3 mm	Yes *b*-value ≥ 800	In distal tumours, a coronal sequence angulated parallel to the anal canal should be included to assess the relation between tumour and anal sphincter
**1**	Fernandez-Martos, 2010 [24]	1.0/1.5 T	Yes 3 mm	Yes	Yes 3 mm	Yes 3 mm	Yes 3 mm	Yes 3 mm	NA	NA	Turbo spin echo
**2**	Dewdney, EXPERT C, 2012 [25]	NA	NA	NA	NA	NA	NA	NA	NA	NA	Thin sliced MRI (3 mm) High resolution
**3**	Jakobsen, 2012 [26]	NA	NA	NA	NA	NA	NA	NA	NA	NA	
**4**	Smith, 2015 [27]	1.5/3.0 T	Yes	Yes	NA	NA	NA	NA	NA	Yes	
**5**	Achiam, 2015 [28]	1.5 T	NA	NA	NA	NA	NA	NA	NA	NA	Slice thickness 5 mm T1WI Contrast enhanced Bowel extension
**6**	Deijen, COLOR III, 2016 [29]	NA	NA	NA	NA	NA	NA	NA	NA	NA	
**7**	Glynne-jones, BACCHUS, 2015 [30]	NA	NA	NA	NA	NA	NA	NA	NA	NA	
**8**	Burbach, RECTAL BOOST, 2015 [31]	NA	NA	Yes	NA	NA	NA	NA	NA	Yes	
**9**	Nahas, 2016 [32]	1.5/3.0 T	NA	Yes	Yes 3 mm	Yes	Yes 3 mm	Yes^a^	NA	NA	
**10**	Haddad, 2017 [33]	NA	NA	NA	NA	NA	NA	NA	NA	NA	
**11**	Singh, 2017 [34]	NA	NA	NA	NA	NA	NA	NA	NA	NA	
**12**	Lee, 2019 [35]	NA	NA	NA	NA	NA	NA	NA	NA	NA	
**13**	Jameson, SPAR, 2019 [36]	NA	NA	NA	NA	NA	NA	NA	NA	NA	
**14**	Deng, FOWARC, 2019 [37]	NA	NA	NA	NA	NA	NA	NA	NA	NA	
**15**	Nougaret, GRECCAR 4, 2019 [38]	1.5/3.0 T	Yes	NA	Yes	Yes	Yes	Yes	NA	NA	
**16**	Bahadoer, RAPIDO 2021 [9]	1.5/3.0 T	Yes	NA	Yes	Yes	Yes ≤ 3 mm	Yes	Yes	Optional	T1WI optional
**17**	Conroy, PRODIGE 23, 2021 [39]	NA	NA	Yes 3 mm	NA	NA	NA	NA	NA	NA	3D MRI
**18**	Akiyoshi, NOMINATE, 2022 [40]	NA	NA	Yes	NA	NA	NA	NA	NA	Yes	
**19**	Chen, 2022 [41]	3.0 T	NA	NA	Yes	Yes	Yes	Yes	NA	Yes	Dynamic contrast-enhanced T1WI
**20**	Ominelli, 2022 [42]	NA	NA	NA	NA	NA	NA	NA	NA	NA	

*ESGAR* European Society of Gastrointestinal and Abdominal Radiology; *T*, Tesla; *MRI* magnetic resonance imaging; *T1WI* T1 weighed imaging

^a^When tumour near or involving the anal canal

## Discussion

This observational study presents results of a quality audit of compliance to the MR protocol requirements in a large randomised multicentre trial. In the RAPIDO trial MRI findings were used as tool to identify the eligible patients for inclusion. Out of the 729 patients who were considered for review, data was available for 668 (91.6%) patients, mostly referring to the baseline setting. Of the 1291 MRIs available for review in both settings, only 632 (49.0%) fulfilled the image acquisition requirements concerning slide thickness and MRI sequences as stipulated in the protocol.

**Fig. 4:**
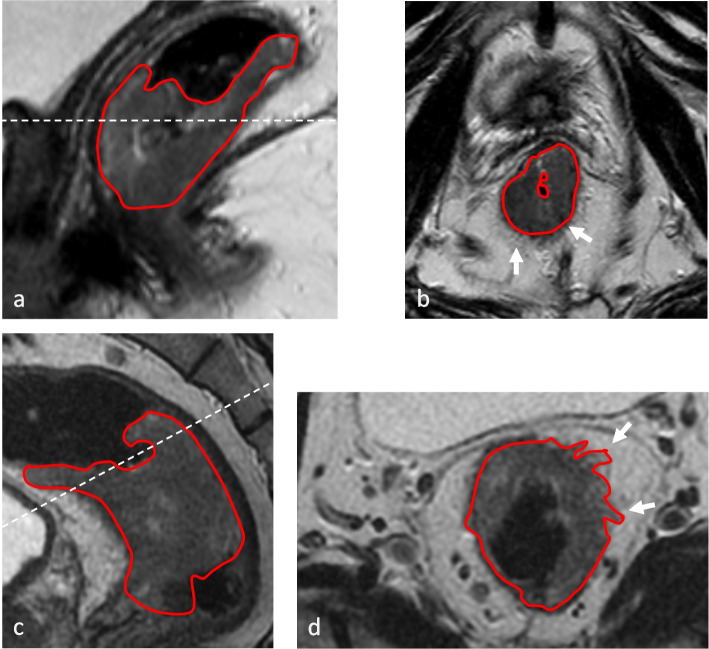
**a**–**d** Sagittal and axial (oblique) T2-weighted MR images from two patients from different centres. Both these investigations were performed in the baseline setting; therefore, differences in image quality are irrespective of the effects of neoadjuvant treatment. Tumour borders are delineated with continuous red lines. White dashed line in **a** and **c** = plane of the axial MR image shown in **b** and **d**. **a**, **b** Both the sagittal and the axial sequences had a slice thickness of 4 mm. In both images, the rectal wall is not clearly defined. **b** Axial projection of the tumour. Structures in the mesorectal fat are not clearly visible. The image of the invasive front is blurred (arrows). No sequence perpendicular to the tumour was obtained for this patient. **c**, **d** Both the sagittal and the axial oblique sequences had a slice thickness of 3 mm. In both cases, the rectal wall is clearly defined, and invasion of the mesorectal fat is distinct. **d** The invasive front is indicated by white arrows

Potential consequences of non-adherence to the protocol include interpretation errors that may result in both over- and under staging [12–15]. Firstly, neglection of high-resolution T2-weighted sequences, images with limitations with respect to signal to noise [12] or a slice thickness exceeding the size of lesions [4, 13–16] induce radiologists to over-estimate the tumour extent [12, 13]. Additionally, a poor angulation as shown in Fig. 4a and b limits the evaluation of the muscularis propria, its relation to the mesorectal structures and therefore the accuracy of the T-stage assessment [12]. Moreover, high-resolution T2-weighted images perpendicular to the tumour’s long axis allow a better detection of extramural venous invasion, one broadly recognised independent predictor of local recurrence, nodal and distant metastases [17]. Similarly, mesorectal fascia (MRF) invasion is a predictor of local recurrence [18] and is considered a criterium for defining locally advanced rectal cancer. While there is sufficient consensus regarding macroscopic invasion of MRF (i.e. margin of 0 mm), agreement decreases when the distance between the tumour and the MRF is ≤ 1 mm (defined as involved MRF) or 1–2 mm (defined as threatened MRF) even with adequate MR images [19]. Low-resolution T2-weighted MR images can interfere with the assessment of MRF invasion. In Fig. 4, MR images that do not fulfil the technical quality criteria of thickness and angulation are compared with a correctly performed investigation. In the case of the RAPIDO study suboptimal quality of baseline MRI could potentially cause incorrect inclusion, while in daily clinical practice, it could lead to inadequate treatment stratification. Similarly, inadequate restaging MRI might lead to inaccurate assessment of the surgical approach [16, 20, 21], resulting in suboptimal oncological outcome [11] and also a potential risk of not detecting a clinical complete response. Additionally, a higher interobserver variability has been reported when assessing MR images that do not fulfil the international guidelines [13]. Therefore, defining and complying to a standard MRI protocol as outlined in international guidelines is of great importance.

The results of this study highlight challenges in multicentre studies, especially when diagnostic imaging is pivotal. Similarly, this heterogeneity in protocols also characterises common clinical practice. In this study, although only a proportion of all imaging performed was reviewed, MR images of in total 40 centres from three countries were reviewed. Even though a well-defined MRI protocol was available, only less than half of the registered MRI examinations fulfilled the quality criteria. In particular, there was a trend showing that for the MRI scans of centres that mostly did not follow the study protocol the reasons for non-compliance to the MR protocols were consistent, suggesting that institutions did not adapt their protocol but kept following their internal MR protocols (Fig. 3a, b). Before start of the RAPIDO study, sites were invited to workshops, but attendance was not obligatory. In future multicentre studies, obligatory workshops should be carried out before the initiation of the study and adherence to the study MR protocol for each participating centre should be assessed before entry of their first patient. Also, regular audits throughout the study period should be performed ensuring the quality of MRI in all centres. An example of systematic quality assessment that is often performed prior to inclusion of the first patients in clinical trials in radiotherapy is the so called dummy run. The implementation of quality requirements is hereby assessed in each participating centre and major discrepancies are solved prior to initiation [22, 23]. Similarly, investigators of future RCTs where MRI plays a pivotal role could consider performing mandatory audits of MR investigations performed in each centre prior to patients’ randomisation. To address this problem in common clinical practice, all centres should be aware of and follow the most recent ESGAR guidelines [4].

Out of the 20 RCTs used for comparison, 11 (55%) reported some MRI requirements, all fulfilling the recommendations of the 2016 ESGAR consensus meeting that was published in 2018 [4]. However, no information was reported regarding how many of the MRI examinations followed the defined protocol. To our knowledge, the RAPIDO is the first RCT for patients with locally advanced rectal cancer that carried out a quality assessment of the imaging performed within the study.

This study has several limitations. Firstly, only 89.5% of the expected MRIs and 69.0% of the examinations from the whole study were assessed for technical features of image acquisition. This was partly explained by the substantially different imaging storing and sharing systems across the participating institutions. For the future, compatible sharing systems enabling easy image retrieval during and after trials are much desired. Secondly, the image quality was assessed by two separate reviewers with limited clinical experience in MRI, and this study mainly reviewed the technical parameters specified in the MRI files and explicitly required in the study protocol. Consequently, other relevant aspects regarding imaging quality such as field of view and voxel size, matrix, suboptimal surface coil placement, wrap-around and motion artefacts, signal-to-noise issues or artefacts related to metallic implants or air were not specifically considered in the audit. Additionally, no DWI quality parameters were assessed. All these aspects play a paramount role in a thorough evaluation of MR images’ quality, but these requirements were not specified in the study protocol and therefore not evaluated in this audit. Lastly, the inclusion of patients for the RAPIDO study, so strongly dependent on MRI criteria, started in June 2011, more than 2 years prior to the publication of the first ESGAR consensus guidelines [8] and 7 years before the publication of the current guidelines [4]. At the time of first inclusions, the international quality recommendations for MR imaging were therefore less clearly defined. However, the MRI acquisition criteria stipulated in the RAPIDO protocol (see Additional file 1: Supplementary Materials) are entirely in line with the current ESGAR guidelines [4].

In conclusion, this quality audit of MR acquisition protocol in a large multicentre rectal cancer trial shows that a significant proportion of examinations were not performed in accordance with the study protocol. Besides having important impact on inclusion and treatment of patients in the study, the results highlight the importance of proper trial preparation including radiology. Additionally, simultaneous systematic centralised image quality control during large clinical trials, when feasible, can contribute to more appropriate patient inclusion and treatment.

### Supplementary Information

**Additional file 1:** **Supplementary Materials**.

## Data Availability

The data used for this audit is stored in a secured drive in the servers of the Leiden University Medical Centre, Leiden, the Netherlands. Data management and storage has been coordinated by the local Clinical Research Centre. Access to this data is limited and can be granted to the members of the study group upon request.

## Abbreviations

DWI: Diffusion-weighted imaging
ESGAR: European Society of Gastrointestinal and Abdominal Radiology
LARC: Locally advanced rectal cancer
MR(I): Magnetic resonance (imaging)
MRF: Mesorectal fascia
RAPIDO: Rectal Cancer And Pre-operative Induction Therapy Followed by Dedicated Operation
RCT: Randomised controlled trial
TME: Total mesorectal excision
