# Supplementary material for: Results of a diagnostic imaging audit in a randomised clinical trial in rectal cancer highlight the importance of careful planning and quality control
Source: Insights Imaging. 2023 Nov 24;14:206. doi: 10.1186/s13244-023-01552-0 (PMC10673763; doi:10.1186/s13244-023-01552-0)
Supplement: Supplementary file 1 — Additional file 1: Supplementary Materials. [file 13244_2023_1552_MOESM1_ESM.docx]

Appendix G - Magnetic resonance imaging

# Requirements

1.5T or 3T equipment

Phased-array receiver coils for pelvic-/body imaging No obligatory preparations.

(Four hours fasting) allowed Antispasmodic agents allowed

No endoluminal or intravenous contrast agents

*Image sequences*

T2-weighted high resolution sequences in at least three different planes (Sagittal, transaxial and oblique planes) where one imaging sequence is perpendicular to the rectum at the level of the tumour interleaved with maximum 3 mm section thickness (1). If low tumours, additional oblique sequences including the tumour parallel and perpendicular to the anal canal are performed.

T1-weighted axial images of the pelvis and diffusion weighted images of the pelvis allowed but can only be performed if the quality of the T2-weighted images have been guaranteed.

# MRI reporting

*Level of the tumour*

The distance of the tumour from the anorectal junction and/or from the anal verge is measured by electronic calipers on sagittal images. The length of the tumour is measured and reported. It is also stated whether the tumour is above, at or below the level of the peritoneal reflection. For low tumours it is stated whether the tumour is within a mm from the levator muscles or not, whether there is involvement of the intersphincteric plane and the external sphincter.

*Morphology*

of the tumour is described whether the tumour is polypoid, (semi)annular. If there is evidence of a mucinous tumour indicated by typical high signal intensity on T2-weighted images, this is also reported.

*Depth of extramural spread*

The maximum depth of extramural depth from the outer edge of the muscle layer to the outer edge of the tumour is measured on high resolution images perpendicular to the rectum at the level of the tumour (1)

*Extramural vascular invasion*

is recorded when there is tumour extension along a vessel as a serpintiguous extension of tumour signal within a vascular structure (2)

*Mesorectal Fascia*

Involvement of the potential circumferential margin is defined as tumour extending within 1 mm of the mesorectal fascia or closer.

*Perforation of the peritoneal reflection by tumour*

Is reported when nodular extension of tumour beyond the peritoneal reflection is found (3)

*Mesorectal lymph node metastases*

The total number of mesorectal lymph nodes is reported and the number of lymph nodes regarded as metastatic according to morphological criteria by G. Brown et al. A suspicion of mesorectal lymph node metastases is high if two or more of the following morphological criteria can be appreciated on high resolution T2-weighted sequences – round, irregular border and heterogeneous signal intensity (4).

In addition to the morphological criteria. The following criteria are also used

Mesorectal lymph nodes with a short axis diameter of more than 10 mm and round shape are regarded as metastatic. Mesorectal lymph nodes between 5-9 mm and at least two of the ciriteria round shape/irregular border/heterogeneous signal intensity are also regarded as metastatic.

*Extramesorectal lymph node metastases*

Presence of suspected metastatic inguinal, lateral pelvic lymph nodes should be reported. Metastatic extramesorectal lymph nodes or pelvic sidewall lymph nodes are defined by morphological criteria similar as for mesorectal lymph nodes: irregular border and/or heterogeneous signal intensity and/or round (not-oval) lymph nodes with short axis diameter of more than 10 mm.

*Evaluation post chemoirradiation*

When MRI is performed after neoadjuvant treatment, the post treatment MRI is compared with MRI at baseline. Viable tumour (high signal intensity) is separated from post treatment fibrosis (low signal intensity) on T2-weighted images. For mucinous tumours, remaining or increasing pure mucin pools may not necessary indicate progressive disease.

Length of tumour and tumour and fibrosis if these are not clearly separated is measured as in baseline on sagittal T2-weighted images.

The minima distance of tumour (and fibrosis) is measured and the transaxial direction noted 1-12 O´clock.

Regarding lymph nodes, the short axis diameter of mesorectal and extramesorectal lymph nodes is measured. Lymph nodes with malignant morphological features pre-treatment and a short axis diameter post treatment of equal to or more than 5 mm are considered malignant.

A clinical complete response (CR) or near CR is defined as presence no visible tumour on T2- weighted images with normal bowel wall layers or presence of residual fibrosis that is confined to the bowel wall.

**References:**

1. Taylor FG, Swift RI, Blomqvist L, Brown G.

A systematic approach to the interpretation of preoperative staging MRI for rectal cancer. AJR Am J Roentgenol. 2008 Dec;191(6):1827-35. Review.

1. Smith NJ, Shihab O, Arnaout A, Swift RI, Brown G. MRI for detection of extramural vascular invasion in rectal cancer. AJR Am J Roentgenol. 2008 Nov;191(5):1517-22. Review.
2. Brown G, Radcliffe AG, Newcombe RG, Dallimore NS, Bourne MW, Williams GT. Preoperative assessment of prognostic factors in rectal cancer using high-resolution magnetic resonance imaging. Br J Surg. 2003 Mar;90(3):355-64.
3. Brown G, Richards CJ, Bourne MW, Newcombe RG, Radcliffe AG, Dallimore NS, Williams GT. Morphologic predictors of lymph node status in rectal cancer with use of high-spatial-resolution MR imaging with histopathologic comparison. Radiology. 2003 May;227(2):371-7.
4. Engelen SM, Beets-Tan RG, Lahaye MJ, Kessels AG, Beets GL. Location of involved mesorectal and extramesorectal lymph nodes in patients with primary rectal cancer: preoperative assessment with MR imaging. Eur J Surg Oncol. 2008 Jul;34(7):776-81.
5. Lahaye MJ, Beets GL, Engelen SM, Kessels AG, de Bru√Øne AP, Kwee HW, van Engelshoven JM, van de Velde CJ, Beets-Tan RG. Locally advanced rectal cancer: MR imaging for restaging after neoadjuvant radiation therapy with concomitant chemotherapy. Part II. What are the criteria to predict involved lymph nodes? Radiology. 2009 Jul;252(1):81-91.
6. MERCURY Study Group. Relevance of magnetic resonance imaging-detected pelvic sidewall lymph node involvement in rectal cancer. Br J Surg. 2011 Sep 16.
